# Periprosthetic joint infections – a scoping review

**DOI:** 10.1515/iss-2024-0016

**Published:** 2024-11-20

**Authors:** Yasmin Youssef, Annika Hättich, Kim Lydia Klepka

**Affiliations:** Klinik für Orthopädie, Unfallchirurgie und Plastische Chirurgie, Universitätsklinikum Leipzig, Leipzig, Germany; Department of Trauma and Orthopaedic Surgery, University Hospital Hamburg Eppendorf, Hamburg, Germany; Orthopädische Klinik, Stiftung Herzogin Elisabeth Hospital (HEH), Braunschweig, Germany

**Keywords:** arthroplasty, periprosthetic joint infections, diagnostic and treatment algorithms, standards, quality

## Abstract

Periprosthetic joint infections (PJIs) are dramatic complications after primary total joint arthroplasty. Despite increasing research in this field, the diagnosis, classification, and management of PJI remain a challenge. This is mainly due to the heterogenous clinical presentation of PJI in clinical routine and patient-related factors as secondary diagnosis and periprosthetic tissue condition. The early detection of PJI is essential for adequate treatment. However, there is no definition for PJI with 100 % sensitivity or a negative predictive value. This can potentially lead to a delayed or missed diagnosis of PJI. Furthermore, the surgical and antibiotic treatment is among the most discussed topics in PJI literature. There is no international consensus on the treatment of different PJI entities. Concludingly many aspects of PJI diagnostics and treatment remain controversially discussed and current studies are only comparable to a limited extent due to study heterogeneity and limited comparability.

## Introduction

Periprosthetic joint infections (PJIs) are rare but dramatic complications after primary total joint arthroplasty of the knee and hip 1], [2], [3. Next to aseptic loosening they are among the most frequent causes for revision arthroplasty. The incidence is 1–3% after primary hip and knee arthroplasty [4, 5]. The total incidence is however thought to be even higher because of undiagnosed low-grade infections as well as gaps in reporting. In revision arthroplasty reinfection rates can be as high as 16 % 6], [7], [8.

PJIs do not only pose a severe socioeconomic burden, but also are disabling for the patient, as they are associated with more complex treatment intervals, more frequent and longer in-hospital stays, more invasive treatment and longer periods of immobilisation [3, 9]. In addition to that patients with PJIs have high mortality rates as well as higher morbidity and a reduced quality of life 1], [2], [3.

Despite the continuous and intensified interest and research in this field, the diagnosis and management of PJIs remain a challenge. Diagnosis can be complicated as PJIs can show similar symptoms as aseptic loosening, especially low-grade infections [10, 11]. On the other hand, improperly treated or even missed PJIs can lead to persistent infections, with associated reduction in quality of life and increased morbidity [6, 12, 13]. The treatment of PJIs consists of an interdisciplinary approach with a combination of surgical and antibiotic interventions and screening for potential secondary foci when appropriate [6, 12, 13]. Even if different recommendations for the management of the PJI have been published, many aspects of PJI treatment remain controversially discussed [6, 12], [13], [14.

The aim of this scoping review therefore is to present new insights gained of literature published in 2023–2024 on the diagnosis, classification, and treatment of PJI.

## Methods

A scoping literature research of all studies published on PJI between 01/2023 and 03/2024 on PubMed was performed using the search-term “periprosthetic joint infection”. A total of 1,389 articles were found. The authors formulated four subtopics “periprosthetic joint infection and definition”, “periprosthetic joint infection and classification”, “periprosthetic joint infection and antibiotic treatment” and “periprosthetic joint infection and surgical treatment”, to narrow the search. Case reports, case series, opinion papers, technical notes, scoping reviews and articles written in languages other than English or German were excluded. In addition to that studies that were irrelevant or unrelated to the four subgroups were excluded. After title screening a total of 327 articles were identified for abstract evaluation. All articles were independently assessed by two of the authors. In total 10 articles were chosen for discussion in this scoping review [8, 14], [15], [16], [17], [18], [19], [20], [21], [22. All included articles were discussed by all authors and the information was comprehensively summarised.

## Definition

There are various definitions used for the diagnosis and definition of PJIs (Table 1). The most common ones are the definitions proposed by the Musculoskeletal Infection Society (MSIS), the Infectious Diseases Society of America (IDSA) and the International Consensus Meeting (ICM) 23], [24], [25], [26. The latest definition was published by the European Bone and Joint Infection Society (EBJIS) in 2021 [23]. The EBJIS definition includes the following criteria: Clinical appearance, blood workup with C-reactive protein (CRP), synovial fluid cytological analysis and biomarkers, microbiology, histology, and nuclear imaging and distinguishes between three categories: 1. infection unlikely (all criteria negative), 2. infection likely (two positive criteria) and 3. infection confirmed (one positive criteria) [23].

**Table 1:** Comparison of present PJI definitions.

McNally et al. EBJIS (2021) [23]	Schwarz et al. ICM (2019) [25]	Parvizi et al. MSIS (2011) [24]	Osmon et al. IDSA (2013) [26]
Clinical features and blood workup (CRP) Synovial fluid cytological analysis and biomarkers – Leucocyte count with PMN% – Alpha-defensin Microbiology – Aspiration fluid – Interoperative samples (fluid and tissue) – Sonication Histology – High-power field (400x) Other – Nuclear imaging	Major Criteria: – Sinus tract – Two positive cultures for the same pathogen from different samples (tissue or fluid) Minor criteria: – Preoperative: – Elevated serum markers (CRP, D-dimer, ESR) – Elevated synovial markers (CRP, PNM%, alpha-defensin, WBC or LE) – Intraoperative: – Preoperative score – Positive histology – Purulence – Single positive culture	Major criteria: – Sinus tract – Two positive cultures for the same pathogen from different samples (tissue or fluid) Minor criteria: – Elevated serum CRP and ESR – Elevated synovial fluid WBC OR change (++) on leukocyte esterase test strip – Elevated synovial fluid PMN% – Positive histological analysis of periprosthetic tissue – Single positive culture	Criteria: – Sinus tract – Purulence – Acute inflammation in histology – Two positive cultures (same pathogen) OR one positive culture with high virulent organism

**Scoring system**			

– Infection unlikely (all findings negative) – Infection likely (two positive findings) – Infection confirmed (one positive findings)	1 major criterium OR Sum of minor criteria: – ≥ 6: Infected – 2–5: Possibly infected – 0–1: not infected	1 major criterium OR ≥ 4 of the minor criteria	≥ 1 positive criteria

CRP, C-reactive protein; PMN%, Percentage of polymorphonuclear neutrophils; ESR, erythrocyte sedimentation rate; WBC, white blood cell count; LE, Leukocyte esterase.

In 2023, Sousa et al. published a multicentric validation study, in which the definitions were compared in terms of sensitivity and negative predictive value [15]. The EBJIS definition showed the highest rate of cases categorized as infected and compared to the other definitions showed the highest rate of culture-negative infections (EBJIS: 28 %, ICM: 19 %, IDSA: 19 % and MSIS: 9 %). In addition to that the study showed that in comparison to older classifications the EBJIS is more sensitive in the identification of PJIs. The perioperative sensitivity is 89 % (CI: 84–93) for the EBJIS definition and 85 % (CI: 79–90) for the ICM definition. Similarly, the negative predictive value (NPV) was shown to be higher for EBJIS (90 %; CI: 85–93) than it is for ICM (87 %; CI:82–90) [15]. Table 2 presents the sensitivity and negative predictive value of the different PJI definitions (EBJIS, ICM, IDSA, MSIS).

**Table 2:** Comparison of sensitivity and negative predictive value of the different definitions (EBJIS, ICM, IDSA, MSIS).

Definition	Sensitivity	NPV
EBJIS	89 % (CI: 84–93)	90 % (CI: 85–93)
ICM	85 % (CI: 79–90)	87 % (CI: 82–90)
IDSA	56 % (CI: 48–65)	77 % (CI: 74–80)
MSIS	66 % (CI: 57–74)	85 % (CI: 81–88)

Similarly Sigmund et al. showed in their retrospective single-centre study with 206 cases that the EBJIS definition, when compared to the ICM and IDSA definitions, has the highest sensitivity [27].

Concludingly, there is no definition for PJI with 100 % sensitivity or negative predictive value. Potentially this can lead to a delayed or missed diagnosis of PJI, which can produce persistent infections, which are more complicated to treat [6, 12, 13]. Concludingly the diagnosis of PJIs depends largely on the definition used, which in turn has a direct impact on patient treatment [28]. The EBJIS definition is currently the most accurate definition, with the highest sensitivity and negative predictive value, for the diagnosis of PJI [15]. Still, different definitions of PJIs used in research with varying degrees of accuracy make it more difficult to compare studies. In addition, the definitions do not differentiate between various PJI entities (early infections, hematogenous/acute late and chronic infections), even if it was previously shown that they differ significantly in their laboratory and microbiological presentation and seem to have different clinical outcomes [14].

## Classification

PJI are usually classified as acute, delayed or chronic infections and are based on the onset of symptoms i.e. clinical presentation after implantation [29, 30]. Different time periods have been described for the different PJI entities with no international consensus [12, 14, 29, 30]. The PJI entity seems to have an impact on the clinical early outcomes with respect to reinfection, mortality and the occurrence of complications [14]. Due to the heterogenous nature of PJI, customized PJI therapy remains a challenge and there is an urgent need to expand and specify the clinical description of PJI.

In 2020 Alt et al. presented the PJI-TNM classification which is based on the well-known TNM classification used in oncology and was adapted to PJIs [31]. In this classification “T” stands for “Tissue and implant conditions”, “N” for “non-human cells (bacteria and fungi)” and “M” for “Morbidity of the patient” in relation to the Charlson Comorbidity Index. The classification is further complemented by coding the affected joint (h for hip, k for knee, s for shoulder), and the presence of a reinfection (r) at the beginning of the code [31].

A retrospective cohort study by Lunz et al. compared the clinical applicability and the correlation between the PJI-TNM classification and clinical outcomes and variables [17]. These included amongst others the course of the initial operation (duration, total blood loss, bone loss), mortality, type of spacer used, re-implantation rate and the rate of unplanned revisions were compared. A total of 80 chronic PJIs, according to the MSIS/ICM definition, after total knee arthroplasty were analysed. To ensure better feasibility in the clinical setting, the TNM classification was modified and simplified (PJI-pTNM). The authors concluded that both classifications enable reliable predictions about the invasiveness of the operation, the possibility of a re-implantation and patient mortality. Factors that significantly influence the clinical outcomes in terms of reinfection rate (r-Status) were the type of spacer used, the duration of the initial operation and bone loss. The tissue and implant conditions (T-Status), the type of surgery, “re-implantation (yes/no)” and the bone and blood loss during the initial operation had a significant influence on the clinical outcome. Finally, the patient’s preoperative condition (M-status) should also be classified, either according to the Charlson Comorbidity Index or the American Society of Anaesthesiologists (ASA).

Classifications have long been used to describe PJI in clinical practice [29, 30], but they only differentiated between acute or chronic, which is not sufficient, as they only emphasize the temporal aspect of the diagnosis and do not consider the heterogeneity of PJI presentation and individual local and patient condition in clinical practice. More distinctive classifications, such as the pTNM-classification should therefore be developed and used in research and clinical practice to improve patient care.

## Treatment

The treatment of PJIs consists of an interdisciplinary approach which combines surgical and antibiotic interventions [6, 12, 13]. PJI therapy is highly dependent on many patient-related and non-patient related factors which can influence the outcome. Factors include the onset of the infection (acute or chronic), type and status of the implanted prosthesis, general health condition of the patient and the type of pathogen detected [14, 32], [33], [34. When applicable, secondary foci should be screened for and eradicated [6, 12, 13]. Different recommendations for the management of different PJI entities have been published [6, 12], [13], [14. Nevertheless, many aspects of PJI treatment remain controversially discussed.

### Surgical treatment

Current concepts in the surgical treatment of PJI include debridement, antibiotics, and implant retention (DAIR), one-stage and two-stage revision. The infection classification, the present pathogen, the condition of the periprosthetic tissue, as well as the presence of comorbidities and potential secondary foci play a role in the chosen surgical method [6, 12, 13]. DAIR is usually used in patients with early infection, infections with non-biofilm producing pathogens and patients with no prosthesis loosening [6, 12, 13, 35]. Two-stage revision is usually still recommended in case of chronic infections [6, 12, 13]. As one-stage exchange has potential advantages like a shorter treatment duration, faster rehabilitation, psychological benefits and socioeconomic benefits, the comparison of one- and two-stage changes is of growing interest [7, 36].

A meta-analysis by Zhao et al. including 40 studies with a total of 8,711 patients discovered that there is no significant difference between one-stage and two-stage revision in terms of reinfection and reoperation rates [18]. However, the authors critically discussed there is a great heterogeneity in existing literature. Inconsistencies in the PJI entities included, specified exclusion and inclusion criteria, the definition of reinfections, as well as varying lengths of follow-up strongly affect the comparability of the studies. In addition, the authors noted a dominance of non-randomised studies, introducing allocation bias.

In two-stage revision reimplantation of the prosthesis is only recommended when the infection has been fully eradicated [37]. In case of persistent infections further debridement and spacer exchanges are recommended [37]. Trampuz and Zimmerli initially defined 2–4 weeks as short and 6–8 weeks as long intervals to implantation [38]. Other authors however recommended other intervals and there is a great heterogeneity in the length of reimplantation intervals reported in clinical studies [39, 40]. A recent review by Puetzler et al. suggested that shorter intervals to reimplantation might show comparable or even superior clinical results when compared to longer intervals [19]. This could be explained by the fact that spacers act as foreign material which bacteria can recolonize as soon as the function of the drug eluting spacer decreases. Furthermore, shorter intervals to reimplantation can also lead to better outcomes due to shorter immobilisation periods [19].

A current review by Sousa et al. investigated the current possibilities in determining the time of reimplantation in two-stage revision [16]. The review demonstrated that up to date there is still no validated and fully reliable diagnostic method for the determination of infection eradication and thus also the optimum timing for reimplantation. While biomarkers like differential cell count and alpha-defensin have become established in the diagnosis of PJI, limited sensitivity and accuracy was shown for the diagnosis of persistent infection. Similarly, there remains limited data on the use of leukocyte esterase for the identification of persistent infection. There is also no validated cut-off for the number of total leukocytes and polymorphonuclear neutrophils in the definition of persistent infections. It was further shown that “antibiotic holiday periods” - antibiotic free period before reimplantation – before reimplantation remain controversially discussed and there are no clear recommendations for their application [16].

### Microbiological treatment

Periprosthetic joint infections are mainly caused by biofilm causing pathogens, also referred to as ESKAPE organisms, which include *E. faecium, Staph. aureus, K. pneumoniae, A. baumannii, P. aeruginosa,* and Enterobacter species 41], [42], [43. Biofilms protect pathogens against natural immunological defence mechanisms and antibiotic treatment 41], [42], [43. The most frequent pathogens isolated in PJI are *Staph. aureus, Staph. epidermidis* and *P. aeruginosa* 41], [42], [43. Biofilm active antibiotics are referred to as antibiotics that have the capacity to penetrate biofilms and act on the embedded bacteria. Further requirements for antibiotics used in PJI treatment are adequate bone penetration capacity and good bioavailability [44]. Usually Rifampicin, as a biofilm active antibiotic, is used for methicillin-sensitive staphylococcal infections in combination with another antibiotic (for example fluoroquinolones) to prevent the development of resistances [20, 45]. In case of methicillin resistant staphylococcal infections Vancomycin and Daptomycin in combination with rifampicin, minocycline or linezolid are mostly used [20, 45]. It must however be noted that different antibiotic schemes might be used in different regions.

Within periprosthetic joint infection treatment the length of antibiotic treatment is among the topics discussed most controversially. Not only the answer of the optimal total duration of antibiotic therapy remains unclear, but also whether different infection entities and surgical treatments (e.g. DIAR, one-stage or two-stage exchange) require different antibiotic treatment concepts.

In a meta-analysis by Olearo et al. which included 10 studies with a total of 1,389 patients (range per study: 37 to 404; 748 THA, 638 KHA, 3 other), it could be shown that there was no significant difference in short and long interval antibiotic treatment [21]. Short interval antibiotic treatment was defined as antibiotic treatment for 4 to<12 weeks (n=604), while long interval antibiotic treatment was defined as > 12 weeks of treatment (n=759). The study included 4 RCTs and 6 retrospective cohort studies. The surgery performed was two stage exchange in five studies, DAIR in 4 studies, one stage exchange in one study. Five out of 10 studies had a low risk of bias and all except for one study had at least one year of follow-up. Overall, there was an 11 % lower risk for treatment failure in short interval antibiotic treatment. There was however no significant difference, even when stratified according to the surgical treatment performed. The pooled odds ratio favoured short interval antibiotic treatment in patients treated with DAIR and long interval antibiotic treatment for patients treated with one- or two stage exchange [21].

Yen et al. found no difference between short and long interval antibiotic therapy in patients treated with DAIR [46]. On the other hand, a current RCT by Bernard et al. demonstrated a higher risk of treatment failure in patients treated with 6 weeks of antibiotics, than 12 weeks [47]. The risk of failure was especially high for patients treated with DAIR [47]. Similarly, the RCT by Lora-Tamayo showed non-inferiority of short antibiotic regimens (8 weeks of treatment) compared to long regimens (minimum of 3 months of treatment) in patients treated with DAIR and staphylococcal infections [48].

Overall long treatment intervals of over 12 weeks total duration are still recommended, especially for patients who have undergone a prosthesis-preserving surgical procedure with DAIR. The treatment duration might however be shortened in cases of low risk of failure [20].

There is also an increasing body of literature that highlights the effects and negative aspects of long-term antibiotic treatment. A current prospective study by Reinecke et al. assessed the adverse events associated with the prolonged antibiotic treatment in periprosthetic joint infections [22]. The study included 80 patients with periprosthetic joint infections of the hip, knee, or shoulder joint. Both patients with acute and chronic infections as well as patients that received, one-stage replacement, two-stage replacement and DAIR were included in the study. Patients received antibiotics for a mean of 12 weeks (range 4–168 weeks). Only 9 % of the included patients reported no occurrence of adverse events. In total 336 adverse events occurred in 73 patients, which led to therapy adjustment in 29 % of the patients and early discontinuation in 6 % of the patients. The most frequent adverse events were loaded in the gastrointestinal tract (46 %), skin and skin appendage (22 %) and the nervous system (14 %). Patients treated with Rifampicin had a higher occurrence rate of adverse events. No significant difference was found in the occurrence of adverse events when comparing sex and patient age [22].

## Conclusions

Concludingly many aspects of in the comprehensive handling of PJI including diagnostics and treatment remain controversially discussed. The following scoping review has highlighted that current studies are only comparable to a limited extent. The definitions for reinfection and treatment failure as well as the inclusion and exclusion criteria and the lengths of the follow-up periods vary significantly between the studies. The treatment of PJIs consists of an interdisciplinary approach which combines surgical and antibiotic interventions. When applicable, secondary foci should be screened for and eradicated. The length of required intravenous antibiotic treatment is still discussed controversially. Furthermore, many studies do not seem to differentiate between different PJI entities, which could lead to significant biases. Large, prospective multicentre studies and RCTs are long overdue and needed to establish adjusted treatment standards for patients.
